# Insights into the Black Box of Intra-Amniotic Infection and Its Impact on the Premature Lung: From Clinical and Preclinical Perspectives

**DOI:** 10.3390/ijms23179792

**Published:** 2022-08-29

**Authors:** Ying Dong, Stefano Rivetti, Arun Lingampally, Sabine Tacke, Baktybek Kojonazarov, Saverio Bellusci, Harald Ehrhardt

**Affiliations:** 1 Department of General Pediatrics and Neonatology, Universities of Giessen and Marburg Lung Center (UGMLC), German Center for Lung Research (DZL), Justus-Liebig-University, Feulgen Street 12, 35392 Giessen, Germany; 2 Cardio-Pulmonary Institute (CPI), Universities of Giessen and Marburg Lung Center (UGMLC), Justus-Liebig-University, Aulweg 130, 35392 Giessen, Germany; 3 Clinic for Small Animals (Surgery), Faculty of Veterinary Medicine, Justus-Liebig-University, Frankfurter Street 114, 35392 Giessen, Germany; 4 Institute for Lung Health (ILH), Universities of Giessen and Marburg Lung Center (UGMLC), German Center for Lung Research (DZL), Justus-Liebig-University, Aulweg 130, 35392 Giessen, Germany

**Keywords:** intra-amniotic infection, prenatal, bronchopulmonary dysplasia, premature infant, inflammation, animal model

## Abstract

Intra-amniotic infection (IAI) is one major driver for preterm birth and has been demonstrated by clinical studies to exert both beneficial and injurious effects on the premature lung, possibly due to heterogeneity in the microbial type, timing, and severity of IAI. Due to the inaccessibility of the intra-amniotic cavity during pregnancies, preclinical animal models investigating pulmonary consequences of IAI are indispensable to elucidate the pathogenesis of bronchopulmonary dysplasia (BPD). It is postulated that on one hand imbalanced inflammation, orchestrated by lung immune cells such as macrophages, may impact on airway epithelium, vascular endothelium, and interstitial mesenchyme, resulting in abnormal lung development. On the other hand, excessive suppression of inflammation may as well cause pulmonary injury and a certain degree of inflammation is beneficial. So far, effective strategies to prevent and treat BPD are scarce. Therapeutic options targeting single mediators in signaling cascades and mesenchymal stromal cells (MSCs)-based therapies with global regulatory capacities have demonstrated efficacy in preclinical animal models and warrant further validation in patient populations. Ante-, peri- and postnatal exposome analysis and therapeutic investigations using multiple omics will fundamentally dissect the black box of IAI and its effect on the premature lung, contributing to precisely tailored and individualized therapies.

## 1. Introduction

The preterm birth rate remains high at approximately 10% globally despite current preventive measures, presenting a great challenge and concern [1]. Intra-amniotic infection (IAI) is one major driver of preterm birth and occurs in up to 70% of deliveries before 28 weeks of gestational age (GA) [2]. Extremely preterm infants (EPIs) with lung development still in the late canalicular or saccular stage are highly susceptible to perinatal stressors, and IAI has been indicated to exert an impact on neonatal morbidities, especially bronchopulmonary dysplasia (BPD), one of the most common and severe complications of preterm birth [2,3]. However, large meta-analyses in the last decade only demonstrated a moderate magnitude of association between IAI and BPD, and the heterogeneity of literature was substantial [4,5]. Nowadays in the post-surfactant era with the use of protective ventilation strategies, BPD is characterized by arrested alveolarization and aberrant vascular development, with life-long restricted lung function [2,6,7]. Although not fully elucidated, the pathogenesis of BPD is considered to be multifactorial, with inflammation being one principal downstream pathway which may have been initiated by antenatal events such as IAI and aggravated by postnatal factors such as oxygen exposure and mechanical ventilation [6]. To date, effective strategies to prevent and treat BPD are scarce.

Under normal circumstances, the intra-amniotic cavity remains an inaccessible black box during gestation, and the management of EPIs is usually not possible until after delivery. The healthcare of EPIs was drastically optimized during the last decades, including antenatal steroids to promote lung maturity, exogenous surfactant to alleviate respiratory distress syndrome, non-invasive ventilation to reduce volume- and barotrauma of the immature lung, caffeine to improve pulmonary outcome in the short-term and neurodevelopment in the long-term, vitamin A supplementation and early adequate caloric intake to support pulmonary development, as well as the integration of non-medical interventions (e.g., family-centered care) [8,9,10,11,12,13]. However, the incidence of BPD remains high and was not significantly improved in the last two decades [14,15,16]. It has been increasingly recognized that failure to reduce BPD is likely attributed to its multifactorial origin, and the impact of adverse factors in the antenatal period may be difficult to avoid and remain insufficiently tackled [17,18]. To push forward the limits of perinatal-neonatal medicine and further improve the pulmonary outcome of EPIs, an in-depth pathomechanistic understanding of BPD directed towards its early origins in the prenatal period is necessary. To achieve this aim, dissection of the impact of IAI on the premature lung is central. Clinical investigations, although abundant, were heterogeneous in this issue. So far, preclinical animal studies have not sufficiently focused on infection in the particularly vulnerable antenatal period, and studies simulating prenatal infection with the investigation of pulmonary sequences in the postnatal period are lacking. This narrative review aims to summarize relevant clinical and preclinical data that provided insights into the black box of the intra-amniotic cavity and explored molecular, structural, and functional changes in the premature lung following infection within a vulnerable time window. 

## 2. Intra-Amniotic Infection: Microbes, Maternal-Fetal Inflammation, Pathological and Clinical Manifestations

**Figure 1:**
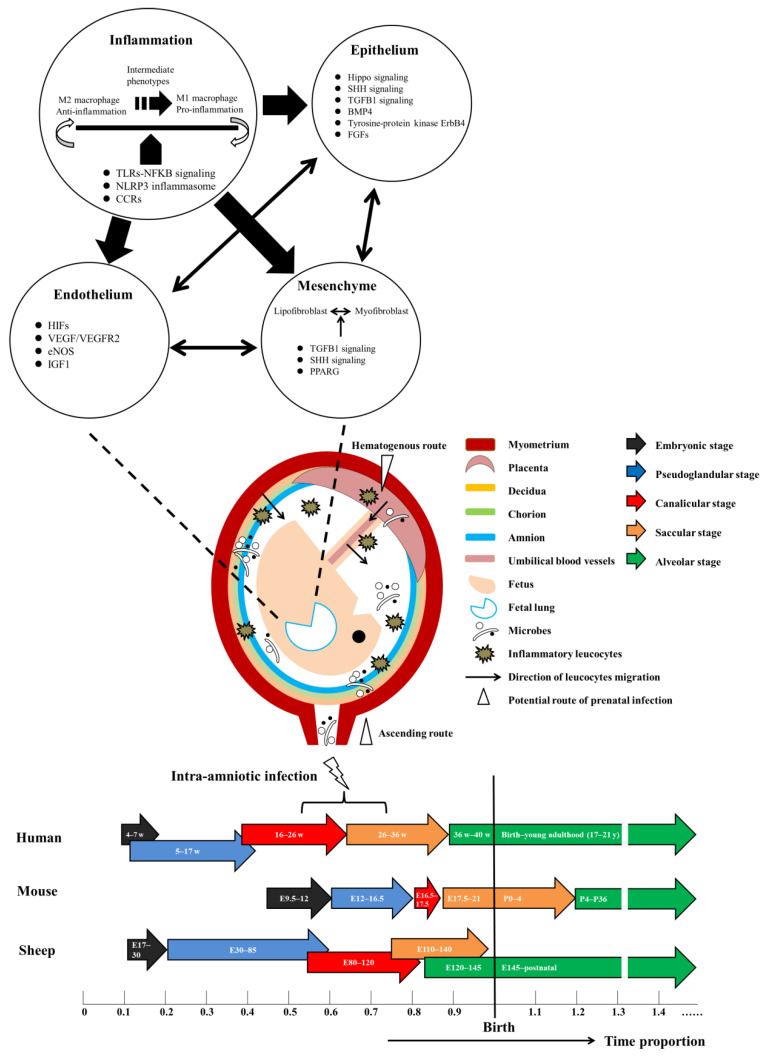
Schematic illustration of prenatal infection between pseudoglandular and canalicular/saccular stage and pulmonary consequences in the three lung compartments. Lung developmental stages between humans and animals which are most frequently used in preclinical studies are compared. The listed potential molecular mechanisms are mainly derived from in vivo animal models focusing on prenatal infection. BMP4: bone morphogenetic protein 4, CCRs: C-C motif receptors, eNOS: endothelial nitric oxide synthase, FGFs: fibroblast growth factors, HIFs: hypoxia-inducible factors, IGF1: insulin-like growth factor 1, NFKB: nuclear factor kappa B, NLRP3: NLR family pyrin domain-containing protein 3, SHH: sonic hedgehog, TGFB1: transforming growth factor beta 1, TLR: toll-like receptor, PPARG: peroxisome proliferator-activated receptor gamma, VEGF: vascular endothelial growth factor.

The conventional notion that the intrauterine environment is sterile has been fundamentally challenged by advances in molecular microbiological techniques. Accumulating evidence pointed towards a role of dysregulated host–microbe relationship in the pathogenesis of preterm birth and neonatal morbidities [19,20,21,22,23,24,25]. Microorganisms, such as *Ureaplasma* and *Mycoplasma* spp., Gram-positive and -negative bacteria, fungi, and viruses, were detected in varying abundance and taxonomic diversities at different sites of the maternal-fetal interface (e.g., chorioamnion and amniotic fluid) [19,20,21,22,23] (Figure 1). Ascending invasion of vaginally colonized microorganisms into the intra-amniotic cavity [20,21], with hematogenous dissemination being debated as an equally important route [20], are considered to be major sources of in utero microbial exposure [24,25]. 

Invading microorganisms can elicit maternal and/or fetal inflammatory responses, characterized by infiltration of maternal leucocytes from decidua to chorioamnion and/or fetal leucocytes from umbilical blood vessels to Wharton’s jelly, respectively, and finally reach amniotic fluids [26,27,28] (Figure 1). The migration of leucocytes was shown to follow chemotactic gradients of inflammatory mediators, especially interleukin 6 (IL6), a marker widely used to define intra-amniotic inflammation [27,29]. Strictly speaking, chorioamnionitis and funisitis are pathological diagnoses of IAI from the maternal and fetal side, respectively [26]. Each of them can be classified into different stages and grades based on the location and intensity of neutrophil influx [26]. It should be noted that an inflammatory response with enhanced IL6 levels in amniotic fluids has been documented both in the presence and absence of microbial invasion [29,30,31,32]. As such, Triple I criterion “intrauterine inflammation, infection, or both” was recently recommended to better describe this broad disease spectrum [33]. By evaluating microbiota and pro-inflammatory cytokines in amniotic fluids and placenta, the published studies hinted towards a dose-dependent relationship between the magnitude of intra-amniotic inflammatory response and neonatal morbidities [23,31]. In terms of clinical manifestation, mothers with chorioamnionitis are mostly asymptomatic, and those exhibiting fever plus other signs (i.e., leukocytosis, purulent cervical fluid, maternal, and/or fetal tachycardia) may have normal placenta histopathology [34,35]. 

The discordance and overlap of microbial, inflammatory, pathological, and clinical profiles of IAI underscore a complicated host-microbe interaction during gestation. Modulating factors may include: (1) the load and composition of maternal microbiota [23], microbial type (e.g., high virulent Gram-negative bacteria and relatively low virulent *Ureaplasma* spp.) [19], pathogenic factors of microorganisms (e.g., toxin, biofilm-forming capacity) [36], and microbial exposure in a sequential manner [2]; (2) maternal pro- and anti-inflammatory immune status is dynamically regulated across gestational trimesters to tolerate the semi-allogenic fetus while confronting potential pathogens and preparing to trigger delivery [37]; (3) the developing fetal immune system is not less able to elicit inflammatory responses upon microbial exposure, as compared to their term counterparts and adults, but may incline to imbalanced hyper-inflammation [38,39]. 

## 3. Intra-Amniotic Infection and BPD: Discrepant Patient-Based Biosample and Clinical Evidence

Except for rare circumstances where amniocentesis is indicated, the intra-amniotic cavity remains inaccessible. As such, intrapartum and postpartum exposome analyses of tracheal aspirates and blood samples from preterm infants are instrumental to retrospectively assess prenatal infection and predict neonatal outcome. Patients who were exposed to chorioamnionitis and went on to develop BPD may contain elevated levels of inflammatory cells (e.g., macrophages and neutrophils) and cytokines and chemokines (e.g., IL6, tumor necrosis factor, IL1B, IL10, C-C motif ligand 2, 3, 4, and 5, C-X-C motif ligand 2 and 10), and reduced growth factors (e.g., fibroblast growth factor 10, vascular endothelial growth factor) in their serum and tracheal aspirates collected at birth and in early postnatal life [40,41,42,43,44,45,46,47]. In line with patient-based biosample studies, the most recent and comprehensive meta-analysis recruiting 244,096 preterm or very low birth weight infants suggested an overall association between chorioamnionitis and BPD [4], supporting the hypothesis that perinatal inflammation is implicated in BPD pathogenesis. However, the association between chorioamnionitis and BPD was no longer significant when BPD was further stratified into mild, moderate, and severe BPD [4]. Similarly, there was no difference between infants exposed to chorioamnionitis with funisitis and those with only chorioamnionitis in the risk of BPD [4]. Given the prominent inter-study heterogeneity [4,48,49,50], this again emphasizes that the development of BPD is a multiple-hit process, and the association between IAI and BPD may be modulated by concomitant and sequential perinatal factors.

On the contrary, there are studies failing to identify a correlation between chorioamnionitis and cytokine levels at birth [51]. Some even found less infiltration of inflammatory cells in tracheal aspirates of chorioamnionitis-exposed preterm infants than in those of non-exposed healthy controls [52]. Counterintuitively, preterm infants with lower levels of tumor necrosis factor (TNF), TNF-related apoptosis-inducing ligand (TRAIL), and inflammatory cells in tracheal aspirates at birth may have a higher risk of BPD [52,53,54]. Some clinical studies of infants <34 gestational weeks showed that mild but not severe chorioamnionitis facilitated lung maturity by reducing respiratory distress syndrome [55,56]. Taken together, despite the potential link between perinatal infection, inflammation, and BPD, a certain degree of inflammation may exert benefits on the developing lung within a particular time window. A dynamic balance between pro- and anti-inflammatory responses is necessary for normal lung development, and excessive suppression of inflammation may as well cause lung injury. 

## 4. Prenatal Infection and Its Impact on Lung Development: Representative Animal Models 

Due to limited access to the intra-amniotic cavity and difficulties to control the confounding factors impacting on BPD in clinical studies, preclinical animal experiments are indispensable and ideal to dissect pathomechanisms of IAI-driven lung injury at the tissue, cellular, and molecular level. Representative animal models simulating prenatal infection with short- and long-term pulmonary outcome are summarized as follows.

### 4.1. Rodent Models of Prenatal Infection-Induced Lung Injury

#### 4.1.1. Establishment of Models

Mice and rats most commonly used within experimental studies have a gestational duration of 18.5–21 days and 21–23 days, respectively [3] (Figure 1). Rodents born at term are at the saccular stage, which is structurally similar to the lungs of extremely preterm infants approaching 28 weeks of GA but without the need for supplemental oxygen [3]. Lipopolysaccharide (LPS) has been most frequently used to induce prenatal infection in rodents, via an intraperitoneal (i.p.), intrauterine (i.u.), or intra-amniotic (i.a.) route [40,44,57,58,59,60,61,62,63,64,65,66,67,68,69,70,71,72,73,74,75,76,77,78] (Table 1). Few rodent studies applied viable microorganisms, such as *Ureaplasma* spp. and group B *Streptococcus* (GBS) [79,80]. Recent studies chose ultrasound-guided i.a. injection of LPS, which demonstrated superiority in resembling subclinical prenatal infection over the conventionally applied route such as i.p. injection and intrauterine injection after laparotomy, including enhanced maternal and offspring survival and a low-grade maternal systemic reaction [60,81]. One novel preclinical model using vaginal inoculation of GBS replicated IAI caused by ascending bacterial invasion [80].

In rodent models, fetal exposure to infectious stimuli was mostly induced between the late canalicular and saccular stage [58,59,60,65,68,69,70,71,72,73,74,80], and a few were as early as in the pseudoglandular stage [40,61,62,63,64,66,67,79], mimicking acute and prolonged prenatal infections, respectively. The serotype and dose of LPS vary widely among studies, with the latter ranging from 1 to 10 µg/amniotic sac for rats and 100 pg to 4 µg/amniotic sac and 0.08 to 1 mg/kg i.p. for mice, respectively [40,57,58,59,60,61,62,63,64,65,66,67,68,69,70,71,72,73,74]. In addition, the i.a. injection volume of LPS varied between 5 and 200 µL/amniotic sac [57,58,59,71,72,74]. This represents an important but largely underappreciated confounding factor. There are dynamic changes in the amount and viscosity of amniotic fluid during rodent pregnancy, which may influence the contact between LPS and the fetus, thereby influencing LPS-induced lung injury [82]. Strikingly, most current models demonstrated a high rate of perinatal and postnatal mortality post infection [57,58,59,60,61,62,63,64,68,69,70,73,74], contradicting the clinical fact that the majority of preterm infants survive despite exposure to chorioamnionitis. In general, lower LPS doses, less invasive infection routes, and a shorter duration of infection were associated with improved fetal and neonatal survival [63,67,74]. From these preclinical results, it becomes clear that rodent models intended to study BPD outcome differ from the clinical situation of preterm infants, especially with regard to the severity of IAI.

#### 4.1.2. Maternal and Fetal Inflammatory Response to Prenatal Infection

As early as 3 h after i.p. LPS, cytokines in the maternal serum and amniotic fluid were almost simultaneously enhanced [70]. Based on cytokine levels, the maternal systemic inflammatory response was more pronounced than the localized response after i.p., while the opposite was observed when LPS was given i.a. [60,70], indicating a relevant role of the placenta barrier in the pathogenesis of prenatal infection. Regardless of the injection route, infiltration of inflammatory cells in the decidua and fetal membranes, either concomitantly or sequentially [83,84,85], were evident within 8 h to 4 d post infection [57,63,70,79], signifying the establishment of LPS-induced chorioamnionitis. From the fetal side, 3–8 h following i.p. LPS, TNF, and C-C motif ligand 2 (CCL2) were simultaneously elevated in fetal and maternal serum, with fetal response being weaker than that of the mother [70]. Fetal pulmonary expression of pro-inflammatory cytokines, such as TNF, IL1B, IL6, and CCL2, as well as the influx of neutrophils and macrophages, were observed several hours post LPS and continued to rise until P5–P8, but normalized beyond P14 [44,61,62,64,74,78]. This suggests an acute and transient nature of the pulmonary pro-inflammatory response in mice, driven by prenatal exposure to LPS. 

#### 4.1.3. Molecular, Morphological and Functional Changes of the Lung

In response to inflammatory cytokines, oxidative stress markers (e.g., superoxide dismutase), proteinases (e.g., matrix metalloprotease 9), growth factors (e.g., transforming growth factor beta 1, fibroblast growth factors), arginine-related metabolites, and vascularization-related mediators (e.g., vascular endothelial growth factor, actin alfa 2) were found to be changed at transcriptional and translational levels [57,58,59,61,62,64,65,68,75,76]. Studies performing longitudinal investigation showed persistent regulation of some abovementioned mediators up to the young adulthood period (P21) [64], indicating a sustained impact of prenatal infection on pulmonary molecular signature even after the recession of acute-phase inflammation. As a result, alteration in the three lung compartments of epithelium, endothelium, and mesenchyme may ensue. Larger, fewer, and simplified alveoli were detected as early as P1, being more apparent in the next 1–2 postnatal weeks and persisting up to adulthood [57,58,59,68,69,73,76]. Additionally, vessels became sparse and thick-walled with the loss of endothelial cells and deposition of collagen in the mesenchyme, exhibiting a fibrotic phenotype in histopathology and in microCT [58,59,61,62,64,68,69,71,72,75]. In the meantime, lung resistance increased whereas the compliance, inspiratory capacity, and vessel reactivity decreased between P8 and P21 [64,65,68,69,74]. Some rodent models further reproduced long-term sequelae of BPD, demonstrating significant and prolonged growth retardation from the first postnatal week to adulthood [58,59,68,69,73]. Of note, exposure to LPS or *Ureaplasma* spp. increased the number of alveolar type 2 (AT2) and endothelial cells, enhancing the expression of surfactant proteins (SPs) [40,67,71,72]. Other rodent models did not demonstrate significant changes in lung structure or function in the long term [61,62,64,66]. This is in line with clinical data showing that prenatal infection accelerated lung maturity under certain conditions [56], pointing towards both injurious and protective effects of perinatal inflammation on the premature lung. Variations in rodent strains and the source, duration, and dose of infectious stimuli may underlie inter-study heterogeneity in molecular, structural, and functional alterations of the lung.

### 4.2. Sheep Models of IAI-Induced Lung Injury 

#### 4.2.1. Establishment of Models

Different from rodents but similar to humans, sheep born at term (~E145) are in the alveolar stage [3] (Figure 1). As such, most experimental models delivered lambs per cesarean section at E125, corresponding to ~31 weeks of human gestation [86,87,88,89,90,91,92,93,94,95,96,97,98] (Table 2). The performance of sheep experiments was restricted to several research groups in the USA, Australia, and the Netherlands, thereby ensuring a high degree of homogeneity in animals and the route of prenatal infection, which was exclusively i.a. injection under ultrasound guidance. Moreover, multiple and sequential infectious stimuli (i.e., LPS, IL1, *Ureaplasma* spp.) were given at the pseudoglandular and/or canalicular stage, better simulating prolonged and acute IAI of a polymicrobial nature as encountered in real clinical scenarios [86,87,88,89,90,91,92,93,94,95,96,97,98,99,100] (Table 2). There was no maternal mortality or intrauterine fetal death reported in sheep models, and the birth weights of IAI-exposed preterm lambs were similar to their non-exposed counterparts of the same GA [94,98,100]. In general, ovine models of prenatal infection were more clinically relevant and less heterogeneous in experimental design as compared to rodent models. 

#### 4.2.2. Maternal and Fetal Inflammatory Response to IAI

After exposure to i.a. LPS, ewes developed a localized pro-inflammatory reaction within 5 h to 48 h, marked by elevated cytokines (e.g., TNF, IL1B, IL6, and IL8) and infiltration of inflammatory cells in chorioamnion at first and then in amniotic fluid [91,100]. This simulated the pathological progression of chorioamnionitis in humans [26,27]. Intra-amniotic cytokine levels were observed to peak at 72 h and sustained at a lesser extent up to 45 d after LPS exposure [91,94]. Parallel with the initiation of inflammatory responses in amniotic fluids, expression of cytokines increased almost simultaneously in cord blood and fetal lung within 24 h of LPS injection [90,91,100]. Inflammatory cell influx resolved in the serum of preterm lambs 7 d after IAI but persisted in the lung up to 65 d [94,95], indicating sustained pulmonary inflammation induced by IAI in sheep.

#### 4.2.3. Molecular, Morphological and Functional Changes of the Lung Post IAI

The influence of IAI on developing sheep lung was dependent on the type, duration, and dose of infectious insults. LPS of higher concentrations (4 and 10 mg/sac vs. 0.1 and 1 mg/sac) and a shorter duration (2 d vs. 7 d) elicited more profound cytokine responses and recruited more inflammatory cells in the lung [86,87,88,89,91,92,94]. On the contrary, *Ureaplasma parvum* (UP) provoked none or only mild inflammatory responses, due to their low virulence [93]. Of note, repeated LPS exposure significantly inhibited cytokine elevation in the fetal lung, suggesting LPS tolerance [88,89,95]. Similarly, extensive chronic UP infection in the pseudoglandular stage mitigated subsequent LPS-stimulated fetal pulmonary inflammation [93]. On the contrary, moderately chronic UP infection in the canalicular stage aggravated LPS-induced lung injury, whereas acute UP exposure shortly before delivery did not influence the effect of the subsequent LPS [87,92].

IAI exerted its impact on the premature ovine lung in a spatial manner. While chronic UP infection in the canalicular stage (E83) primarily impaired stem/progenitor populations in the distal airways, an acute LPS insult in the saccular stage (E118) suppressed stem/progenitor cells throughout the entire lung [86]. Depending on the dose and combination of infectious insults, UP and/or LPS could disrupt the expression of vascular endothelial growth factor (VEGF), angiopoietin 1 (ANG1), tunica interna endothelial cell kinase 2 (TIE2), and transforming growth factor beta 1 (TGFB1), predisposing to vascular remodeling and fibrosis [86,87,91,93,96]; meanwhile, the synthesis of SPs and molecules implicated in branching morphogenesis, such as fibroblast growth factor 10 (FGF10) and bone morphogenetic protein 4 (BMP4) were upregulated, resulting in a net beneficial effect as demonstrated by increased lung gas volume [86,87,92,93]. This indicates differential responses of the three lung compartments upon exposure to IAI, and disruptive alterations in the lung can be counteracted by protective responses, leading to a favorable net effect.

In agreement with clinical evidence and rodent models, IAI was found to promote lung maturation in sheep. Ovine models further delineated that only LPS within a certain concentration range (i.e., between 4 and 20 mg/sac) were able to upregulate SPs and saturated phosphotidylcholine [87,90,91,94,95]. There seems to be a linear correlation between fetal lung inflammation and maturation [91,98], and the lung maturity-promoting effect of LPS may not be generated early in the pre-viable period [100]. Following lung maturation driven by LPS, some animals showed enhanced pulmonary mechanics with minimal changes in morphometry [90,91,94,96,98,100], whereas others had reduced alveolar numbers and fewer and thicker pulmonary vessels [86,87,92,95], indicating that early-term benefits of IAI may be obtained at the cost of long-term lung injury. Interestingly, disturbed alveolarization at the cannalicular stage (E100) was observed to be gradually restored at near term (E138) after the cessation of continuous i.a. LPS exposure, suggesting self-recovering potential of the developing lung within a certain time window [94]. Unlike rodent models, there are few data on postnatal pulmonary consequences beyond the neonatal period in ovine models, possibly due to the low *ex utero* survival of preterm lambs in the absence of postnatal intensive care support with mechanical ventilation.

### 4.3. Other Animal Models

Other preclinical models using rabbits, pigs, and non-human primates were in relatively smaller numbers, but recaptured some important findings observed in rodent and sheep models [101,102,103,104]. There is an increasing trend in the last five years to use translational large animal models, such as pigs and Rhesus monkeys, to simulate the real-life intensive care received by EPIs, including but not limited to antenatal steroids, surfactant, caffeine, protective ventilation strategies, and nutritional support [102,103,104]. These highly advanced models are particularly suitable to perform mechanistic studies of preterm birth-associated morbidities and to explore novel therapeutic approaches [104].

## 5. Signaling Networks Underlying IAI-Driven Lung Injury and Potential Targets for Therapeutic Approaches

Due to the scarcity of fetal lung biosamples, the cellular and molecular process underlying IAI-driven premature lung injury was largely investigated in mouse models using loss-of-function and gain-of-function lineages. Other experimental animals (e.g., sheep) are unavailable for transgenic modulation, therefore less advantageous for mechanistic and therapeutic investigations. To date, there is a lack of mouse models reproducing infection which starts in the antenatal period, and our current understanding derives largely from newborn mice exposed to postnatal LPS and/or hyperoxia. These results cannot be extrapolated to lung injuries following prenatal infectious insults, as the underlying signaling events are distinct from those regulated by oxidative and/or inflammatory stress in the context of postnatal lung development [38,39]. Therefore, in vivo mice studies of embryonic lung development and prenatal infection, complemented by biosample analyses from human preterm infants developing BPD and ex vivo data derived from human lung cells and tissues in early life, are mainly referred to in this section.

### 5.1. Signaling Pathways Involved in the Initial Inflammatory Response

During infection, pathogen-associated molecular patterns (PAMPs) of microbes can specifically bind to pattern recognition receptors (PRRs), such as toll-like receptors (TLRs), leading to the expression of cytokines and recruitment of inflammatory cells via activation of the downstream nuclear factorkappa B (NFKB) signaling pathway [105]. Other PRRs, such as NLR family pyrin domain-containing protein 3 (NLRP3) inflammasome [84], C-C motif receptors (CCRs), and IL1 receptor [41,44,106], also play an important role in triggering the inflammatory cascade. Intermediate factors, such as receptor-interacting kinase 3 (RIP3) and CCL2 [41,44], were shown to amplify inflammation, while negative regulators such as A20 dampen the NFKB signaling response [88]. Antagonists against receptors and mediators, such as IL1RA, CCR5 antagonist, anti-IL1B, anti-TNF, and RIP3 inhibitor [45,84,106,107] have shown some degree of efficacy in hindering lung alveolar and vascular injury post LPS exposure.

Increasing preclinical evidence indicated that pulmonary immune cells, especially macrophages, may be major responders to microbial insults in early life [38,41,108]. Macrophages were detectable in embryonic mice lungs as early as E10 and increase with GA [109]. In physiological conditions, macrophages supported the development of airway epithelium, vascular endothelium, and mesenchyme, while undergoing a maturation process marked by the gradual shift from M1 pro-inflammatory polarization at birth towards M2 anti-inflammatory polarization in adulthood [38,110,111]. Contrary to the conventional notion that preterm infants are immune deficient, macrophages from human infants’ cord blood or tracheal aspirates as well as newborn mice lungs were capable of eliciting a sustained pro-inflammatory response upon LPS stimulation, strongly suggesting the establishment of immunocompetence in early life with the tendency towards hyper-inflammation during infection [38,39,112]. Macrophages-activated inflammation was shown by the latest transcriptomic analyses to impact on the pulmonary epithelium, endothelium, and mesenchyme [38,113] (Figure 1). Genotypic and phenotypic heterogeneity and dynamic of lung macrophages were indicated to be closely involved in the homeostasis and post-injury repair of developing lung, but this requires further clarification due to limited current data [38,108,111,112].

Furthermore, both preclinical animal studies and patient-based biosample data illustrated the involvement of the adaptive immune system in fetal and neonatal responses to IAI, including but not limited to the activation of antigen-presenting dendritic cells, CD3+ and CD8 + T Cells [60], and CD4 + CD25 + FoxP3 + Treg cells [90], as well as T cell polarization [39]. This may provide one possible explanation for the persistent course of inflammation following IAI as demonstrated by some preclinical studies and clinical biosample data [43,95]. Strategies restoring the balance between pro- and anti-inflammation via regulation of both innate and adaptive immune responses may hold promise to truly reduce inflammation-driven lung injury.

### 5.2. Downstream Signaling of Epithelium, Endothelium and Mesenchyme in Response to Inflammation

Proper lung development is orchestrated by precise regulation of numerous signaling factors involved in the constant crosstalk among the epithelium, endothelium, and mesenchyme, both temporally and spatially. The latter provides a scaffold for epithelial and endothelial development, and harbors niches for cell regeneration in the other two compartments under disease conditions [114]. The initial branching process of epithelium facilitates de novo vasculogenesis as well as angiogenesis, which in turn contributes to further alveolarization [115]. As such, many signaling processes cannot be simply attributed to one certain lung compartment, and the balance of signaling networks among the three lung compartments is important. Untimely over- or under-expression of these molecules following insults may lead to aberrant lung structure and function, or even death in early life.

In the epithelial compartment, major signaling molecules regulating bud initiation and branch elongation include BMP4, Hippo-YAP1/TAZ, NFKB, tyrosine-protein kinase ErbB4, TGFB1, sonic hedgehog (SHH), and FGFs [53,67,75,99,116], as demonstrated by preclinical rodent and sheep models of IAI [64,75,99]. In particular, complete deficiency of TGFB1 and FGF10 led to mortality at birth because of congenital lung aplasia in preclinical mouse models [117,118]. Notably, the expression and functional profiles of TGFB1 and FGF10 were distinct between the early and late lung development of mice, and deficiency in the early stage seems to be more detrimental [118,119]. By enhanced TGFB1 expression following IAI, collagen deposition was found to increase as demonstrated by preclinical sheep and mice models [61,62,64,99], which is in line with the pro-fibrotic role of TGFB1 [120]. Interestingly, mice deficient in TNF and TRAIL also demonstrated an overexpression of TGFB1 and distorted lung structure, strongly suggesting that a delicate balance between inflammation and TGFB1 is required for normal lung development [53,54]. In current preclinical animal models of IAI, the role of FGF10 was seldom explored [78,99]. FGF10 is synthesized by submesothelial cells and binds to fibroblast growth factor receptor 2b (FGFR2B), which was shown to be regulated by TLR2/4-NFKB signaling [121]. Reduced levels of FGF10 were repeatedly illustrated in tracheal aspirates and lung explants of BPD patients [47,114], while transgenic mice overexpressing FGF10 seem to preserve the normal lung structure [122]. In one latest pioneer preclinical study, i.p administration of recombinant FGF10 triggered de novo alveologenesis in newborn mice with pre-existing BPD-like injuries [122]. Despite being a hyperoxia mouse model, it pointed towards the potential of FGF10 as a promising therapeutic approach to be further validated by preclinical IAI models, given that FGF10 is regulated by both infectious and hyperoxia insults [78,99,114,122].

During pulmonary vascular development, VEGF responds to upstream hypoxia-inducible factors (HIFs) and acts primarily on vascular endothelial growth factor receptor 2 (VEGFR2) to promote endothelial growth and angiogenesis, through the downstream endothelial nitric oxide synthase (eNOS) pathway [68,69]. From the late pseudoglandular phase to canalicular phase, there is a gradual shift of the cellular source of VEGF from the mesenchyme to the epithelium, facilitating the parallel alignment of primitive microvasculature to terminal airways [115]. Similarly, insulin-like growth factor 1 (IGF1) contributes to endothelial development by mediating NO production [77]. Reduced VEGF expression and enhanced levels of soluble FMS-like tyrosine kinase 1 (sFLT1), an endogenous inhibitor of VEGF, were found to be associated with IAI and BPD-like injuries in rat models [58,59,69,92]. Therapies augmenting the HIF-VEGF-eNOS pathway prevented placental and pulmonary vascular abnormalities induced by prenatal LPS, restoring alveolarization, normalizing lung function, and preventing right ventricular hypertrophy (RVH) in rats [68,69,77]. Notably, the same intervention was more effective when administrated prenatally than postnatally [68], which emphasizes the importance of early timing of treatments.

In mesenchyme, resident fibroblasts regulate the homeostasis of the extracellular matrix and the maintenance of epithelial and endothelial cell pools [123]. Particularly, one subpopulation of fibroblasts, lipofibroblasts, which express FGF10 and lipid droplets, were demonstrated to beneficially impact on the development of lung epithelium [124,125], whereas myofibroblasts marked by actin alpha 2 (ACTA2) expression may be associated with BPD [126]. TGFB1 and peroxisome proliferator-activated receptor gamma (PPARG) signaling were demonstrated to govern the two-way conversion between lipogenic and myogenic fibroblast phenotypes, with TGFB1 and PPARG favoring myofibroblast and lipofibroblast differentiation, respectively [127]. Macrophages isolated from mice and humans at birth showed upregulated TGFB1 signaling and downregulated PPARG signaling upon LPS stimulation [38,39], suggesting that lipogenic-to-myogenic transition may be one mechanism underlying aberrant pulmonary development post infection. Metformin, initially the first-line antidiabetic drug, was newly found to promote lipofibroblastic differentiation and reverse lung fibrotic remodeling by inhibiting TGFB1 while activating PPARG signaling [127]. Although not an IAI preclinical model, this pilot study pointed towards a potential novel approach to prevent or restore inflammatory lung injury via modulating fibroblast phenotypes.

Cell-based therapies using mesenchymal stem cells (MSCs) and their secretory products have emerged as the spotlight of therapeutic attempts on a variety of lung diseases, including but not limited to BPD [128,129,130]. Indeed, MSCs share many common characteristics with lung resident fibroblasts [123]. MSCs derived from human fetal tissues or rodent bone marrow, as well as their secretome, showed significant benefits in both prophylactic and rescue treatment of BPD in animal models [128,129]. Meanwhile, phase II clinical trials increased to confirm the safety and efficacy of MSCs-based therapies, either for patients who were at risk of BPD or had already established severe BPD [131,132]. Of note, the most recent in vivo preclinical investigations using MSCs or their extracellular vesicles advanced the therapeutic window from the postnatal period to antenatal period, successfully rescuing normal lung epithelial and vascular development after prenatal LPS insult [17,71]. As MSCs possess multipotent capacities in immunomodulation, lung homeostasis, and regeneration, MSCs-based therapies may be more advantageous compared to interventions targeting single mediators, the efficacy of which has been debated on account of the interacting and redundant nature of relevant signaling pathways. Apart from their therapeutic potentials, MSCs provided additional value as prognostic markers of lung diseases. Based on functional and molecular analysis of MSCs obtained from the tracheal aspirates of ventilated preterm infants at birth, increased proliferation index, NFKBp65 accumulation, and reduced ACTA2 expression of MSCs were inversely associated with the severity of BPD, yielding an area under the curve (AUC) of 0.847 [126,133]. For a comprehensive understanding of MSC-based therapies for BPD, the readers are kindly referred to reviews of leading researchers in this field [18,134].

Last but not the least, prenatal exposure to LPS was shown to epigenetically modulate DNA methylation, histone acetylation, and microRNAs (*miRs*) expression in all three compartments of the developing lung [135,136]. Inhibited expression of *miR29b-3p* was associated with increased TGFB1 signaling, contributing to aberrant alveolarization and matrix composition, which were ameliorated by postnatal administration of *miR29b* [135,136] (Table 3). Consistent with the clinical and preclinical data that BPD perturbs postnatal growth, arginine-related metabolic pathways and glucagon-like peptide 1 receptor (GLP1R) were shown to be suppressed by prenatal LPS, and L-citrullin and exendin-4 as their respective agonists were able to mitigate lung injury and preserve postnatal weight gain [60,76].

## 6. Conclusions and Future Clinical and Preclinical Perspectives

Behind the clinically silent facade of most pregnancies, there is ongoing host–microbe interaction in the intra-amniotic cavity and maternal-fetal inflammatory responses may arise, leading to preterm birth and abnormal lung development with the ultimate outcome of BPD. In current clinical practice, IAI is insufficiently recognized and surveilled in a timely manner, and early caffeine administration [8], postnatal application of corticosteroids [137], vitamin A supplementation [11], and azithromycin therapy for *Ureaplasma*-positive neonates [138] are the only pharmaceutical approaches proved to be effective for reducing BPD. To elaborate on what has been taking place inside “the black box” of the intra-amniotic cavity and inspire “out of the box” therapeutic strategies targeting IAI-induced lung injury, future research should be directed to address the following issues:

(1) From the maternal side, dissection of the microbiome across trimesters under physiological and pathological conditions, being integrated with maternal metabolome, proteome, and immunome, provides the most advanced tools to identify patients at risk of or developing IAI [139,140]. Less invasive or non-invasive approaches are highly desired for this particular population. One pioneer preclinical study using a sheep model has demonstrated that the volatile organic compound profile in exhaled breath could successfully differentiate ewes with UP-induced IAI from non-infected ones with an AUC of 0.93 [141]. Timely detection and appropriate surveillance of high-risk pregnancies is the cornerstone of an optimized healthcare concept starting from the antenatal period, where preventive measures and treatment of prematurity-related morbidities are ideally initiated.
(2) From the neonatal side, minimally invasive exposome analyses of biosamples (e.g., cord blood, oral and laryngotracheal secretion, urine) at birth and longitudinally in the postnatal period are valuable for retrospectively analyzing prenatal events and predicting neonatal morbidities [142,143], yet the roles of many identified biomarkers remain to be further clarified. Cutting-edge single-cell technology and systems biology combining multiple omics will greatly contribute to the exploration and delineation of cell-specific interactions and molecular pathways underlying lung development and injury [38,113,144]. Future neonatal management concept needs to focus on a more personalized approach that accounts for perinatal exposure (e.g., microbial type, duration, and severity of IAI), GA, and multi-omic profiling.
(3) In translational preclinical studies, more sophisticated multiple-hit mice models are warranted to expand the current understanding of the pathogenesis of IAI-induced lung injury at tissue, cellular, and molecular levels, given the advantages of transgenic mice. To truly reflect clinically relevant scenarios, future research should include Gram-positive bacteria and fully consider the polymicrobial and sequential nature of IAI. Technical advances in small animal imaging [61,107], lineage labeling [122,127], and spatial multi-omics [144] potentiate the generation of integrative high-resolution atlas of lung development, homeostasis, and disease, thereby revolutionizing the search for therapeutic candidates.

## Figures and Tables

**Table 1:** A comprehensive list of rodent models of prenatal infection and pulmonary outcomes.

Animals	Infection Route	Type and Dose of Microbial Insults	Time of Prenatal Infection	Fetal/Neonatal Outcome *	Molecular Changes of the Lung *	Structural and Functional Changes of the Lung *
Sprague- Dawley rats [44,78]	i.a. after laparotomy	LPS (055:B5) 1 µg/sac or IL1B 0.5 µg/sac	16.5 dpc	Spontaneous delivery 3–4 d after i.a. LPS	**At P1, P3 and P7:** LPS ↑ a total of 28 pro- and anti-inflammatory cytokines and ΜΦ influx, ↑ *Tie2* and *Fgf*, ↑ LOX activity	**At P1, P3, and P7:** Both LPS and IL1B ↓ terminal airspace and the no. of secondary septa, ↑ MLI
Sprague- Dawley rats [57]	i.a. after laparotomy	LPS (055:B5) 1 µg/sac	16.5 dpc	Spontaneous delivery Mortality at birth: 52% Mortality at P7 and P14: 75% Weight at P7 and P14: normal	**At P1:***Fgfr4*, *Vegfr2* and *Hmox1* ↓, IL6 ↑ **At P7** and **P14:** oxidative markers nitrotyrosine and 8-OHdG ↑	**At P7** and **P14:** MLI ↑
Sprague- Dawley rats [58,59]	i.a. after laparotomy	LPS (055:B5) 10 µg/sac	E20 ^#^	C-section at E22 Mortality at birth: 11% Mortality at P2–P14: 57% Weight at P2–P14: ↓	**At P1:** SOD activity and MnSOD ↑ VEGF and VEGFR2 ↓, eNOS unaffected	**At P7** and **P14:** alveolar count and vascular density ↓, MLI and vascular wall thickness ↑
Sprague- Dawley rat [68,69,77]	i.a. after laparotomy	LPS (055:B5) 10 µg/sac	E20 ^#^	C-section at E22 Mortality at birth: 30% Mortality at P2 and P14: 42% Weight at P14: ↓	**At P1:** sFLT1 ↑, VEGF ↓, HIF1A and HIF2A ↓	**At P14:** alveolar count and vascular density ↓, MLI, vessel wall thickness and RVH ↑, Rrs ↑, Crs ↓
WKAH/htm rats [73]	i.a. after laparotomy	LPS (055:B5) 1 µg/sac	E21 ^#^	C-section at E22 Mortality at birth and P7–P70: 11% and 30–40% Weight at P14–P60: ↓		**At P1, 3, 7, 14, 21, 45, and 60:** alveolar surface density ↓ and radius ↑, alveolar numerical density ↓, no abnormal collagen distribution
Sprague- Dawley rats [74]	i.a. after laparotomy	LPS (055:B5) 1 µg/sac	E20 ^#^	Spontaneous delivery 2 d after i.a. LPS. Mortality at birth: 25%	**At P8:** IL1B and ΜΦ count ↑ **At P8 and P14:** TNF and IL6 unaffected	**At P8 and P14:** alveolar counts ↓, Rrs ↑, Crs unaffected
Sprague- Dawley rats [76]	i.a. after laparotomy	LPS (0111:B4) 10 µg/sac	E20 ^#^	Spontaneous term delivery Mortality at birth: 28.9% Weight at P1–P7: unaffected	**At P3 and P7:** GM-CSF ↓, CXCL10 and ARG1 ↑, TNF, IL1B, IL10, VEGF unaffected, arginine and ADMA ↓	**At P3 and P7:** alveolar size and MLI ↑, secondary crests ↓
C57BL/6 mice [60]	i.a. under ultrasound, i.p.	LPS (0111:B4) 100 ng/sac, LPS (055:B5) 10 µg/mouse	16.5 dpc	PTD before 18 dpc: 87.5% Mortality at birth: >85% PTD before 18 dpc: 80% Mortality at birth: >85%	**At 17.5 dpc:***Il1b*, *Il6*, *Ccl2*, *3* and *5*, *Cxcl1* ↑. Cytokine response: i.a. LPS > i.p. LPS	
C3H/HeN mice [61,62]	i.p.	LPS (0111:B4) 80 µg/kg	16 dpc	20% of dams didn’t deliver Weight at 2 w and 4 w: normal Weight at 8 w: ↓	**At 18 dpc:** *Tnf*, *Cxcl1* and *miR29b* ↓, *Tgfb1*, *Il1b* and *Col1a1* ↑ **At P7:** *Il1b*, *Tgfb1* and *Col3a1* ↑ **At P14:** mediators above unaffected **At 4 w:** ΜΦ ↑	**At P14:** septal thickness and Rrs ↑, air space and Crs ↓ **At 4 w:** air space and septal thickness ↑ collagen, lung Rrs and Crs unaffected **At 8 w:** no. alveoli ↓, septal thickness ↑, fibrosis and alveolar simplification in microCT, Rrs and Crs unaffected
LysEGFP-reporter C57BL/6N mice [63]	i.p.	LPS (0111:B4) 0.25 mg/kg or 1 mg/kg	13 or 17 dpc	2× i.p. 1 mg/kg: PTD at 14 and 18 dpc: 30 and 86%, mortality at 14 and 18 dpc: >90% and 60–70%. 2× i.p. 0.25 mg/kg: no PTD	**At 16, 17 and 18 dpc:** neutrophil infiltration ↑ after 2× i.p. LPS 0.25 mg/kg	
C57BL/6 mice [64]	i.p.	LPS (055:B5) 200 µg/kg	16 dpc	Viable litters Weight at P1, 5, 14: normal Weight at P21: ↓	**At P1:***Tlr4*, *Tnf*, *Il1b*, *Il6*, *Tgfb1* ↑ **At P5:** *Il1b* ↑, other mediators unaffected **At P14:** CTGF ↓, ACTA2 ↑ **At P21:** *Col3a1*, *Tgfb1*, *Ctgf* ↑	**At P21:** ASM area ↑, alveolar count unaffected, Rrs ↑, inspiratory capacity and Crs ↓
C57BL/6 mice [65]	i.p.	LPS (source unknown) 100 µg/kg	E18 ^#^	Spontaneous delivery 3 d after i.p. LPS	**At P21:** iNOS and *Tgfb1* ↑, *Tnf*, *Il1b* and *Il6* unaffected	**At P21:** vessel reactivity ↓, airway reactivity unaffected
C57BL/6J mice [66]	i.p.	LPS (0111:B4) 150 µg/kg	E14 ^#^	Viable litters	**At P3:** TNF, IL1A, IL1RA, IL6, CXCL2, CXCR2, CCL4, sICAM1, C5a, TREM1 ↑	**At P28:** alveolar number, size and surface unaffected
BALB/cJ mice [40,67]	i.a. after laparotomy	LPS (055:B5) 100 pg/sac	15 dpc	No PTD Mortality at birth <5%	**At 17 dpc:** TIE2 ↑ **At 18 dpc:** SFTPA and TLR4 ↑, *Tnf* ↑, *Il1b* ↑	
C57BL/6 and FVB mice [70]	i.p	LPS (0111:B4) 25 µg per mouse	16 or 17 dpc	PTD within 17 h of i.p. LPS Mortality at birth: 61%	**8h after i.p.:** *Tlr2*, *Tlr4*, *Sftpd* ↓	
ErbB4 transgenic mice [71,72]	i.a. under guidance of patent blue dye	LPS (0127:B8) 4 µg/sac	17 dpc	C-Section 24 h after i.a. LPS	**At P1:** TNF and ΜΦ influx ↑	**At P1:** mesenchyme volume and septal thickness ↑, alveolar septa ↓, elastic fibers ↓
CD-1 mice [75]	i.u. after laparotomy	LPS (055:B5) 25 µg per mouse	E17 ^#^	PTD before E19: 55% Mortality at P1: 60%	**6 h after LPS:** Hippo signaling (*Yap1*,*Taz*) ↓ **At E18:** *Il1b*, CXCR2 and HIF1B↑, SFTPB and vimentin (endothelial marker) ↓	**At P1:** alveolarnumber ↓, septal thickness ↑
CD-1 mice [79]	i.a. after laparotomy	UP 5000 cfu/sac	E13.5 ^#^	No PTD. Pups’ survival unaffected	**At 17.5 dpc and P3.5:** IL1A, IL1B, IL6, CCL2, CXCL2 and TGFB1 ↑ **At P3.5:** myeloperoxidase ↑	**At P14.5:** MLI unaffected
BALB/c mice [80]	i.vag.	GBS 3 × 10^4^ cells	17 and 18 dpc	No PTD Mortality at birth, P1, P4: 5%, 21% and 40% Weight at birth and P10: ↓		**At P1:** atelectasis, narrow airway lumen, edema and hemorrhage

* Compared with pups not exposed to prenatal infections. ^#^ No information on plug control. ↑: increased, ↓: decreased. ADMA: asymmetric dimethylarginine, ARG1: arginase1, ASM: airway smooth muscle, ACTA2: actin alfa 2, cfu: colony forming unit, CCL: C-C motif ligand, Crs: compliance, COL1A1: collagen type I alpha 1, COL3A1: collagen type III alpha 1, CTGF: connective tissue growth factor, CXCL: C-X-C motif ligand, CXCR: C-X-C motif receptor, dpc: day post coitum, eNOS: endothelial nitric oxide synthase, FGF: fibroblast growth factor, FGFR4: fibroblast growth factor receptor 4, GM-CSF: granulocyte-macrophage colony-stimulating factor, HIF1: hypoxia-inducible factor 1, HMOX1: heme oxygenase 1, i.a.: intra-amniotic, i.p.: intra-peritoneal, i.u.: intra-uterine, i.vag.: intra-vaginal, sICAM1: soluble intercellular adhesion molecule 1, IL: interleukin, IL1RA: interleukin 1 receptor antagonist, iNOS: inducible nitric oxide synthase, LOX: lysyl oxidase, LPS: lipopolysaccharide, MLI: mean linear intercept, PTD: preterm delivery, Rrs: resistance, RVH: right ventricular hypertrophy, sFLT1: soluble FMS-like tyrosine kinase 1, SOD: superoxide dismutase, SFTPA: surfactant protein A, TIE2: tunica interna endothelial cell kinase 2, TGFB1: transforming growth factor B1, TLR: toll-like receptor, TREM1: triggering receptor expressed on myeloid cells 1, UP: *Ureaplasma parvum*, VEGF: vascular endothelial growth factor, VEGFR2: vascular endothelial growth factor receptor 2.

**Table 2:** Representative sheep models of prenatal infection and pulmonary outcomes.

Type and Doses of Microbial Insults	Time of Prenatal Infection	Groups	Molecular Change of the Lung *	Structural and Functional Change of the Lung *
**Single infectious stimulants**				
LPS (055:B5) 10 mg/sac, or IL1A or IL1B (15 µg or 150 µg/sac) [98]	E118	Control 7 d LPS 7 d IL1A (two doses) 7 d IL1B (two doses)	**At E125:** 150 µg IL1A, 150 µg IL1B and LPS ↑ leukocyte influx, ↑ Sat PC and SPs mRNA, inflammatory effect: LPS = IL1A > IL1B	**At E125:** lung compliance and volume ↑, lung function and Sat PC was positively correlated with leukocytes
LPS (055:B5) 10 mg/sac [100]	E92	Control 2 d LPS	**At E94:** *TNF*, *IL1B*, *IL8* and *CCL2* ↑, no effect on anti-oxidant enzyme mRNA, SPs mRNA, *VEGF* and *CTGF*	**At E94:** lung air space unaffected
LPS (055:B5) 5 mg/sac [90]	E122	Control 7 d LPS	**At E129:** IL8 and neutrophils and myeloid cell influx ↑, CD3^+^ T cells and FoxP3 + T_reg_ cells ↑	**At E129:** lung gas volume ↑, desaturated phospholipids ↑, morphometry unaffected
LPS (055:B5) 10 mg/sac [88,89]	E118 and/or E123	Control 2 d LPS 7 d LPS 2 d + 7 d LPS	**At E124 ± 1:** 2 d LPS ↑*TNF*, *IL1B*, *IL6*, *IL8,* and *A20* with lower values at 7 d LPS, 2 d + 7 d LPS hat no effect indicating LPS tolerance. LPS ↓ CAV1, ↑ TGFB1/SMAD2/3, and A-SMase/ceramide,↑HMOX1	
LPS (055:B5) 10 mg/sac [99]	E107 and/or E114	Control 7 d LPS 14d LPS	**At E120:** 7 d and 14 d LPS ↑ *SHH* and its mediators *GLI1* and *GLI2*, 14d LPS ↑ *BMP4*, *FGF10* and *ELN*, 7 d LPS ↓ *BMP4* and *ELN*, both 7 d and 14 d LPS ↓ elastin foli, 14 d LPS ↑ collagen deposition	
LPS (055:B5) 0.1,1,4 or 10 mg/sac [91,96]	5 h, 1, 3, 7 d before E125	Control 5 h, 1, 3, 7 d LPS	**At E125:** dose-dependent effect to ↑ inflammatory cell influx, *TNF*, *IL1B* and *IL8*, SPs mRNA and H_2_O_2_, which peaked at 24–72 h after LPS	**At E125:** 4 and 10 mg/sac LPS ↑ lung gas volume and compliance, other doses didn’t
LPS (055:B5) 20 mg/sac [97]	E110, E118, E121, E123 or E124	Control 1, 2, 4, 7, 15 d LPS	**At E125:** SFTPD↑ 1 d after i.a. LPS and remained at peak up to 7 d, other SPs peaked 2 d after i.a. LPS and persisted in lower level up to 15 d	**At E125:** Sat PC and lung gas volume ↑ 4 d after i.a. LPS and further ↑ up to 15 d
LPS (055:B5) 1 mg/d pumped i.a., or 10 mg/sac 1xper week [94]	E80 to E10, E100 to E128	Control Continuous LPS, Multiple LPS	**At E100:** Continuous 20 d LPS ↑ neutrophil influx, ↓ eNOS and VEGFR2. **At E130:** multiple 20 d LPS ↑ neutrophil influx, ↑ Sat PC, eNOS, VEGFR2, **E138 and E145:** mild inflammation	**At E100:** ↑ Sat PC, ↓ saccule numbers, **At E 130, 138**, and **145:** no lung abnormality
LPS (055:B5) 0.6 mg/d pumped i.a., or multiple 1 mg/sac injection [95]	E80 to E100, or E60, E80, E100	Control Continuous LPS, 25 d, 45 d, 65 d LPS 25 d + 65 d LPS	**At E125:** all LPS groups ↑ inflammatory cell influx, Sat PC and SPs. 25d + 65 d LPS showed no significant impact vs. either alone	**At E125:** continuous LPS ↓ alveolar number, surface and wall thickness, and lung compliance
**Multiple infectious stimuli**				
UP 2 × 10^5^ CCUs/sac, or LPS (055:B5) 10 mg/sac [86,87]	E80 or 83, E118 or E123	Control 42 d/45 d UP 2 d LPS 7 d LPS UP + 2 d LPS UP + 7 d LPS	**At E125:** 2 d LPS or 2 d LPS + UP ↑ *IL6*, *IL8*, *CCL2* and inflammatory cells, UP or 7 d LPS had relatively milder inflammatory effect. Both UP and LPS ↓ P63^+^ basal cells and TTF1^+^ club cells, ↓ SOX9 cells, Ki67+ cells, AT1 and AT2 cells, ↑ SPs, ↓ *TGFB1*, *VEGFR2*, *ANG1.* UP + LPS had synergistic effect than either insult.	**At E125:** Both UP and LPS ↓ vessel density, but ↑ lung gas volume, only UP + 7 d LPS ↑ MLI
UP 2 × 10^5^ CCUs/sac, and/or LPS (055:B5) 10 mg/sac [92]	E70, E92 or E87	Control 24 d UP 2 d LPS 7 d LPS 24 d UP + 2 d LPS 24 d UP + 7 d LPS	**At E94:** LPS and 24 d UP + LPS ↑ CD3^+^ T cell and neutrophils, UP + LPS ↓ *VEGF, VEGFR2*, *ANG1* and *TIE2*, 24 d UP + LPS had synergistic effect vs. either insult, 7 d LPS vs. 2 d LPS ↓ *VEGFR2*, *ANG1* and *TIE2*	**At 94:** UP + 2 d LPS ↑ fibrosis vs. 2 d LPS alone. 7 d LPS vs. 2 d LPS ↑ wall-to-lumen ratio of pulmonary arterioles
UP 2 × 10^7^ CCUs/sac, and/or LPS (055:B5)10 mg/sac [93]	E54 or E 117, E122	Control 7 d UP 70 d UP 2 d LPS 7 d UP + 2 d LPS 70 d UP + 2 d LPS	**At E124 ± 1:** UP alone induced mild inflammatory responses, LPS alone ↑ *IL1B*, *IL6*, *IL8*, and *IL10*, *CCL2*, *IL1RA*, ↑ CD3^+^ T cells, neutrophils and myeloid cells, ↑ TGFB1 Effect: 7 d UP + LPS = LPS alone, 70 d UP + LPS antagonized LPS alone	**At E124 ± 1:** LPS alone ↓ lung gas volume, other groups didn’t

* Compared with prenatal saline exposure if not otherwise indicated. ↑: increased, ↓: decreased. AT1: alveolar type 1, ANG-1: angiopoietin-1, A-SMase: acid-sphingo-myelinase, BMP4: bone morphogenetic protein 4, CAV1: caveolin 1, CCL2: C-C motif ligand 2, CCUs: color changing units, CTGF: connective tissue growth factor, CXCL2: C-X-C motif ligand 2, dpc: day post coitum, eNOS: endothelial nitric oxide synthase, ELN: elastin, eNOS: endothelial nitric oxide synthase, FGF10: fibroblast growth factor 10, HMOX1: heme oxygenase 1, IL: interleukin, LPS: lipopolysaccharide, MLI: mean linear intercept, SHH: sonic hedgehog, TGFB1: transforming growth factor beta 1, TIE2: tunica interna endothelial cell kinase 2, Sat PC: saturated phosphatidylcholine, SFTPD: surfactant protein D, SPs: surfactant proteins, UP: *Ureaplasma parvum*, VEGF: vascular endothelial growth factor, VEGFR2: vascular endothelial growth factor receptor 2.

**Table 3:** Examples of promising strategies to prevent or treat prenatal infection-induced lung injury in preclinical rodent and sheep models in the last 5 years.

Animal Model of IAI (N of Each Arm)	Interventional and Control Arms of IAI	Effect on Fetal and Neonatal Lung (Treatment vs. Control Arm)
**Mice**		
i.a. LPS at 17 dpc [71] N = 4–11	i.a. adult BMSCs or HPSCs control (each 2 × 10^6^ cells) at the same time of i.a. LPS	**At 18 dpc:** BMSCs ↑ lung morphological maturation, but ↓ SFTPC. The effect was more pronounced in the presence of ErbB4 receptor
i.p. LPS at E14 and 65% O_2_ P1–P28 N = 3–20 [106,107]	Postnatal daily s.c. IL1RA 10 mg/kg or saline control 1× per day for 28 d	**At P28:** IL1RA ↓ alveolar size, ↑ alveolar number and surface area, ↓ collagen thickness and ACTA2, ↓ vascular resistance, but no effect on airway hyperreactivity. **At P60:** improved pulmonary vascular structure
i.a. LPS at 16.5 dpc [84] N = 8–10	Dam i.p. NLRP3 inflammasome inhibitor MCC950 50 mg/kg or PBS control 1–2 h before LPS	Preterm delivery before 18.5 dpc and mortality at birth ↓ by 30%
i.u. LPS at E17 [75] N = 11	Dam i.p. melatonin 10 mg/kg or saline control at E17 before LPS #	Preterm delivery before E19 and mortality up to P1 ↓ by about 60%. **At P1:** ↓ IL1B, CXCL2 and septal thickness ↑ SFTPB and endothelial cells
i.a. LPS at 16 dpc [135,136] and 85% O_2_ P1–P14 N = 4–8	Postnatal treatment of intranasal *miR29b* (1 × 10^9^) in viral/lipid vector or vector control on P3	**At P28:** only *miR29b* in viral vectors ↓ septal thickness and showed trend to ↑ alveolarization, ↓ defects in matrix structure
**Rats**		
i.a. LPS at E20 [17] N = 12–20	i.a. MSCs-derived extracellular vesicles (0.25 × 10^6^/sac) or none at E20 at the same time of LPS	**At P14:** treatment restored alveolar and vascular growth, lung function, and RVH to normal level as seen in LPS non-exposed pups
i.a. LPS at E20 [68] N = 10	Prenatally: anti–sFLT1 mAb i.a. 1.5 µg/sac or saline after LPS Postnatally: anti–sFLT1 mAb i.p. (1 or 10 mg/kg), control IgG or saline 2× per week for 2 weeks	Prenatal but not postnatal treatment ↑ neonatal survival from birth up to 2 weeks by about 50% **At P14:** pre- and postnatal treatment ↑ alveolar count, vessel density, lung function, and ↓ RVH
i.a. LPS at E20 N = 8–20 [77]	Postnatal i.p. rhIGF1/BP3 (0.02, 0.2, 2, or 20 mg/kg) or saline control for 2 weeks	**At P14:** eNOS and IGF1 ↑, RAC and vessel density was restored to normal values as in saline control, RVH, Rrs and Crs was improved in a dose-related manner
i.a. LPS at E20 [69] N = 15	Prenatally: DMOG 10 mg/sac or GSK360A 1 mg/sac or saline control after LPS * Postnatally: DMOG 5 mg/kg or GSK360A 5 mg/kg i.p. or saline control every 2 days for 2 weeks	**At P14:** Pre- and postnatal treatment ↑ alveolar count, vessel density, lung function, and ↓ RVH. Prenatal treatment ↑ HIF1A and HIF2A, VEGF, and eNOS, and improved placental structure
i.a. LPS or IL1B at 16.5 dpc, N = 7–8 [44]	i.a. anti-IL1B 0.5 µg/sac, or CCR5 antagonist DAPTA 1 µg/sac, or RIP3 inhibitor GSK872 2 µg/sac, or BTA 0.4 µg (nature product), or saline control at 16.5 dpc	**At P1, P3, and P7:** all drugs ↓ inflammation, ↑ terminal airspace and no. of secondary septa, ↓ MLI
i.a. or i.p. LPS on the dam at 16.5 dpc [60] N = 8–10	i.p. Exendin-4 30 µg/kg or saline control 6 h after LPS	Exendin-4 ↓ preterm deliveries and ↑ neonatal survival from birth to P15 **At P15:** Exendin-4 ↓inflammation in pups with i.a. but not i.p. LPS exposure
**Sheep**		
i.a. LPS at E122 [90] N = 5–7	Fetus received 250,000 IU/kg/d of IL2 or heparinized saline at E118 via umbilical catheter for 4 d before LPS	**At E129:** systemic IL2 did not inhibit inflammatory cell and IL8 responses in fetal lungs, but ↑ lung function

* DMOG and GSK360A are prolyl-hydroxylase inhibitors (PHi) increasing HIF1 expression. # Melatonin was shown to inhibit Hippo pathway. ↑: increased, ↓ decreased. ACTA2: actin alpha 2, BMSCs: bone marrow-derived mesenchymal stem cells, BP3: binding peptide 3, CCR5: C-C motif receptor 5, Crs: compliance, CXCL2: C-X-C motif ligand 2, dpc: day post coitum, eNOS: endothelial nitric oxide synthase, HIF1: hypoxia-inducible factor 1, HPSC: hematopoietic stem cells, IAI: intra-amniotic infection, i.a.: intra-amniotic, i.p.: intra-peritoneal, rhIGF1: recombinant human insulin-like growth factor 1, IL: interleukin, IL1RA: interleukin 1 receptor antagonist, LPS: lipopolysaccharide, *miRs*: microRNAs, MLI: mean linear intercept, NLRP3: NLR family pyrin domain-containing protein-3, s.c.: subcutaneous, sFLT1: soluble FMS-like tyrosine kinase 1, RAC: radial alveolar count, Rrs: resistance, SFTPC: surfactant protein C, PBS: phosphate buffered saline, RIP3: receptor-interacting kinase 3, RVH: right ventricular hypertrophy, UP: *Ureaplasma parvum*, VEGF: vascular endothelial growth factor.

## Abbreviations

ACTA2	actin alpha 2
ANG1	angiopoietin 1
AT2	alveolar type 2
AUC	area under curve
BMP4	bone morphogenetic protein 4
BPD	bronchopulmonary dysplasia
CCL2	C-C motif ligand 2
CCR	C-C motif receptor
CTGF	connective tissue growth factor
CXCL2	C-X-C motif ligand 2
eNOS	endothelial nitric oxide synthase
EPIs	extremely preterm infants
FGF	fibroblast growth factor
GA	gestational age
GBS	group B *Streptococcus*
GLP1R	glucagon-like peptide-1 receptor
HIF1	hypoxia-inducible factor 1
HMOX1	heme oxygenase 1
IAI	intra-amniotic infection
i.a.	intra-amniotic
IGF1	insulin-like growth factor-1
IL	interleukin
IL1RA	interleukin 1 receptor antagonist
i.p.	intraperitoneal
i.u.	intrauterine
LPS	lipopolysaccharide
*miRs*	microRNAs
MSCs	mesenchymal stromal cells
NFKB	nuclear factor kappa B
NLRP3	NLR family pyrin domain-containing protein 3
PAMPs	pathogen associated molecular patterns
PPARG	peroxisome proliferator activated receptors gamma
PRRs	pattern recognition receptors
RIP3	receptor-interacting kinase 3
RVH	Right ventricular hypertrophy
SHH	sonic hedgehog
SPs	surfactant proteins
sFLT1	soluble FMS-like tyrosine kinase 1
TGFB1	transforming growth factor beta 1
TIE2	tunica interna endothelial cell kinase 2
TLR	toll-like receptor
TNF	tumor necrosis factor
UP	*Ureaplasma parvum*
VEGF	vascular endothelial growth factor
VEGFR2	vascular endothelial growth factor receptor 2
